# A review of equity issues in quantitative studies on health inequalities: the case of asthma in adults

**DOI:** 10.1186/1471-2288-11-104

**Published:** 2011-07-12

**Authors:** Heather L Greenwood, Nancy Edwards, Amandah Hoogbruin, Eulalia K Kahwa, Okeyo N Odhiambo, Jack A Buong

**Affiliations:** 1 Institute of Population and Public Health, Canadian Institutes for Health Research, 312-600 Peter Morand Crescent, Ottawa, K1G 5Z3, Canada; 2 Institute of Population Health, University of Ottawa, 1 Stewart Street, Ottawa, K1N 6N5, Canada; 3 School of Nursing, University of Ottawa, 451 Smyth Road, Ottawa, K1H 8M5, Canada; 4 Department of Epidemiology and Community Medicine, University of Ottawa, 451 Smyth Road, Ottawa, K1H 8M5, Canada; 5 Faculty of Community and Health Studies, Kwantlen Polytechnic University, 12666 72nd Avenue, Surrey, V3W 2M8, Canada; 6 The UWI School of Nursing, Mona University of the West Indies, 9 Gibraltar Camp Way, Kingston 7, Jamaica; 7 Department of Research and Knowledge Development, Great Lakes University of Kisumu, P.O. Box 2224, Kisumu, 40100, Kenya; 8 Department of Community Health and Development, Great Lakes University of Kisumu, P.O. Box 2224, Kisumu, 40100, Kenya

## Abstract

**Background:**

The term 'inequities' refers to avoidable differences rooted in injustice. This review examined whether or not, and how, quantitative studies identifying inequalities in risk factors and health service utilization for asthma explicitly addressed underlying inequities. Asthma was chosen because recent decades have seen strong increases in asthma prevalence in many international settings, and inequalities in risk factors and related outcomes.

**Methods:**

A review was conducted of studies that identified social inequalities in asthma-related outcomes or health service use in adult populations. Data were extracted on use of equity terms (objective evidence), and discussion of equity issues without using the exact terms (subjective evidence).

**Results:**

Of the 219 unique articles retrieved, 21 were eligible for inclusion. None used the terms equity/inequity. While all but one article traced at least partial pathways to inequity, only 52% proposed any intervention and 55% of these interventions focused exclusively on the more proximal, clinical level.

**Conclusions:**

Without more in-depth and systematic examination of inequities underlying asthma prevalence, quantitative studies may fail to provide the evidence required to inform equity-oriented interventions to address underlying circumstances restricting opportunities for health.

## Background

Although sometimes used interchangeably, the terms 'health inequality' and 'health inequity' are not synonymous. Inequalities in health are only considered health inequities if they are deemed unjust and avoidable. While inequities in health are inequalities in that they reflect differences in status, capacity, or opportunity that shape risk factors and affect health outcomes, not all inequalities are inequities. The concept of inequity incorporates a values-based decision on whether differential findings by relevant social category (e.g. gender, class, race) are unfair and unjust [1]. Similarly, while 'health disparities' may incorporate inequities, not all disparities are inequitable [2]. These distinctions have important consequences for the way differences in health are understood and interventions are designed and measured [2-4]. Quantitative studies provide essential measures of health status that can inform action on health inequalities. However, a number of authors have suggested that some areas of quantitative research have not adequately discussed or interrogated the equity issues underlying such inequalities [5-7].

### Implications of an equity approach

Through an assessment of historical, political, cultural, and socio-economic context, an equity analysis seeks to identify differences in the risk factor profile or health outcomes between socially advantaged and disadvantaged groups that can be mitigated through policy and resource redistribution approaches. From an equity perspective follows a moral imperative to take action on health inequalities founded in injustice. This is grounded in a strong body of evidence, which shows that socially disadvantaged groups systematically experience worse health outcomes [4]. Thus, an equity perspective shifts from an exclusive focus on proximal issues, such as personal behaviors and lifestyle choices, to "upstream" factors (e.g. economic inequality, social hierarchies) that influence opportunities for health [4]. Understanding pathways to health equity, the proximal to distal factors that interact to produce inequities, is essential for the design of interventions that promote equitable opportunities for health and well-being [8].

### The case of asthma in adults

In light of the growing recognition for placing equity at the centre of population and public health analysis, this review sought to better understand whether or not discussions of equity have permeated quantitative literature about health inequalities and asthma in adults.

Asthma affects approximately 300 million adults and children worldwide, with prevalence rates ranging from 1-18% globally [9,10]. Of notable concern are the increases in asthma prevalence in some African, Latin American and Asian countries [11,12]. Furthermore, there are racial and socioeconomic differences in asthma prevalence, morbidity, and mortality rates [13-16]. Explanations for such inequalities include poverty, variations in environmental and occupational exposures, and differential access to medical care [13,14,17-20]. Reducing the burden of asthma requires efforts to address the underlying conditions that are producing inequities [14].

## Methods

### Search strategy

**Figure 1:**
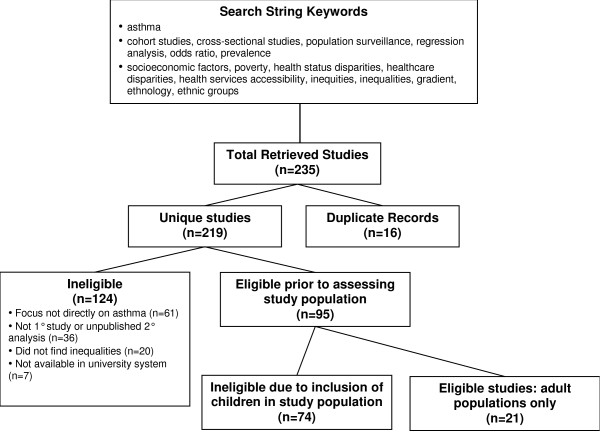
**Relevance assessment of search results**.

Articles were identified based on a systematic search of MEDLINE (Ovid MEDLINE 1950-present) using an *a priori *defined search string (Figure 1). This was developed iteratively through key word identification, testing, and revision. We aimed for high sensitivity to capture a large number of articles that would later be assessed for relevance. All searches took place between June 23 and July 14, 2009.

### Eligibility criteria

Eligibility criteria were: 1) published in English during the years 2005-2009; 2) primary research study or previously unpublished secondary analysis of existing data; 3) outcome variables included health outcomes and/or health care utilization patterns directly related to asthma in adult populations with asthma or at risk of developing asthma; 4) data analysis demonstrated inequalities with respect to the outcome variables measured; 5) analysis of inequalities compared respondents on the basis of SES, and/or gender, and/or race/ethnicity, and/or place of work, and/or place of residence. Multiple articles based on the same data set but with different foci and analyses were included. If the same abstract was retrieved more than once, duplicate abstracts were excluded.

Each abstract was assessed initially for relevance by two independent reviewers (HLG, NE, SD, RN). In cases of disagreement, a third assessment was conducted by a reviewer who had not initially assessed the abstract in question (HLG, AH, RN). If eligibility could not be determined based on the abstract alone, the full article was retrieved for further assessment (HG, NE).

### Data extraction

Basic study information was extracted from eligible articles (e.g. aims, design, study type, location). For 'study type', articles were classified as either etiological/risk factor studies or health services-oriented, as we expected different approaches to studying and reporting on equity issues. 'Etiological/risk factor studies' included not only biological and individual social risk factors such as genetic predisposition to disease, income and educational status, but also social determinants of disease and what Glass and McAtee [21] describe as risk regulators such as employment conditions, transportation corridors and industrial zoning of neighbourhoods. The 'study design' category classified studies as cross-sectional, prospective cohort, or retrospective cohort.

In addition to study information, extraction focused on two types of data: 1) objective data that identified whether or not the terms 'equity/inequity' were used in the study, and described findings and intervention options, and; 2) subjective data that described pathways to inequity implied by the authors even when equity terms were not used directly. Combined, this extraction aimed to describe asthma-related inequalities, determine whether they were explicitly discussed in equity terms, and consider whether equity issues were raised even if not discussed explicitly.

#### Objective data

Objective data were extracted separately by two reviewers for each article (HG, NE, AH, EKK, ONO, JAB). This included: a description of the inequalities identified, interventions and/or research directions suggested by the authors, and a search for equity-related terminology. With respect to inequalities, reviewers were asked to extract: indicators of inequality and quantitative description of social gradients or other inequalities. Although all included studies identified asthma-related inequalities, only statistically significant findings were extracted because we did not expect authors to discuss inequities if they found no significant differences among social groups.

Reviewers also searched each document for instances of equity-related terminology (HLG, AP). The number of occurrences of each of the following terms, excluding those in the reference section, was counted: equity(ies)/inequity(ies), equality(ies)/inequality(ies), and disparity(ies).

#### Subjective data

Subjective information was extracted by a single reviewer (HLG), based on consultations with the research supervisor (NE) and guided by the question: without using the terms 'equity' or 'inequity', do the authors *implicitly *raise equity issues? The definitions of equity used to guide the extraction suggested important questions for unravelling pathways to health equity: What is unfair or unjust in the context of a given society [1]? What can be done to change the social conditions that shape disadvantage [3,22]? While recognizing the subjectivity inherent in our analysis, we aimed to present a set of issues that were illustrative of health inequities when considered carefully in their social, political, and economic contexts.

All text in which the authors implied inequity by highlighting potential unfairness or by otherwise tracing the pathway to inequity was extracted. For example, the following text was extracted from a study: "In addition to social condition being a factor leading to asthma, it may also be that asthma leads to low socioeconomic level. There are also data indicating lower salaries, unemployment, or difficulties in obtaining a promotion for individuals with work related asthma. These findings, taken together with the present results, permit us to propose the theory of a vicious cycle in which work related asthma leads to lower earnings and is also the consequence of lower socioeconomic level in combination with lower educational level [23]^(p698)^." The reviewer (HLG) wrote the following in their explanation: "The authors highlight an inequitable association between lower socioeconomic status and increased risk of work-related asthma... They also suggest a further inequity whereby work-related asthma may deepen poverty by limiting income and employment opportunities."

Only the discussion and conclusion sections of the article were reviewed for the extraction of subjective information, because it is in these sections that authors discuss the implications of their research.

## Results

The search retrieved a total of 235 abstracts, which was reduced to 219 unique studies once duplicate abstracts were excluded. Of these 219 studies, 212 were available in the University of Ottawa library system and retrieved, and 21 of these retrieved studies met the inclusion criteria. The most common reasons for ineligibility were: population not limited to adults; focus not on health outcomes or health care utilization patterns directly related to asthma in populations with, or at risk of developing, asthma (e.g. mental health of parents caring for children with asthma); not a primary research study or unpublished secondary analysis; and analysis did not identify inequalities based on SES, and/or gender, and/or race/ethnicity, and/or place of work, and/or place of residence (Figure 1).

Of the included studies, 13 were etiological/risk factor research, 5 were health-services oriented, and 3 focused on both these areas (for details see Additional file 1: Characteristics of included studies). Fifteen studies used a cross-sectional design, 4 used a retrospective cohort design, and 2 used a prospective cohort design. The majority of studies were conducted in the United States (n = 13); other countries included Australia (n = 1), Brazil (n = 1), Canada (n = 1), Chile (n = 1), China (n = 2), Sweden (n = 1), and the United Kingdom (n = 1).

### Explicit discussion of equity issues

The terms 'equity(ies)' or 'inequity(ies) were not used in any of the studies (Table 1). A search for other terms to describe health differences revealed one study from the United States [15], and a study from Sweden [24] that used 'equality(ies)' or 'inequality(ies)', both in the discussion section of the article. Additionally, five studies used 'disparity(ies)', all of which were conducted in the United States [15,25-28]. In these articles, 'disparity(ies)' appeared in the title, abstract, introduction, and/or discussion sections. Instead of using the equity, inequality, and disparity terms listed above, we found that authors relied on less specific terminology to describe their findings; 17 of the 21 articles described 'differences' in health status or health care utilization, and all referred to 'relationships' or 'associations' between outcomes and socioeconomic or demographic characteristics.

**Table 1:** Number of studies (n = 21) with occurrences of the terms 'equity', 'disparity', and 'inequality'

Terms	0 Occurrences	1 Occurrence	2-5 Occurrences	6-10 Occurrences	>10 Occurrences
Equity(ies)/Inequity(ies)	21 studies (100%)	0 studies	0 studies	0 studies	0 studies

Equality(ies)/Inequality(ies)	19 studies (90.5%)	2 studies (9.5%)	0 studies	0 studies	0 studies

Disparity(ies)	16 studies (76.1%)	2 studies (9.5%)	1 study (4.8%)	1 study (4.8%)	1 study (4.8%)

Note: Number of occurrences does not include those in the reference section.

### Implicit discussion of equity issues

Although none of the studies explicitly used the term 'equity', we found that all but one implied inequity by attempting to trace, at least partial, pathways to inequity. These articles did so by suggesting upstream social conditions that limited opportunities rather than by explicitly labelling disadvantage as unjust. A full list of the identified pathways to inequity is presented in Table 2.

**Table 2:** Potential pathways to inequity identified among eligible studies

Category of Equity Issue	Example
Access to care	Underdiagnosis of asthma in the Alaska Native population related to a lack of health care providers [35].
	Job requirements that may limit the ability to access health care services during normal business hours [36].

Access to transportation	The role of access to public or private transportation in influencing ambulance service and emergency room use for asthma [30,36].

Discrimination	Potential variability in likelihood of asthma diagnosis due to different standards applied to different ethnic groups [15].

Environmental & occupational exposures	Association between lower educational level, poverty, visible minority status, ethnicity, and/or immigrant status and higher risk of developing asthma due to increased exposure to irritants and occupational risk factors [23,27,32].
	Proximity of low-income urban neighborhoods to sources of pollutants (e.g. highways and truck routes) [39,42]. Potential migration of high-risk populations to areas with lower pollutants [40], and potential reduced exposure to air pollutants in rural areas [41].

Housing & neighborhood environment	Poor housing conditions in low-income neighborhoods that may increase exposure to cockroach and rodent allergens, dampness, and mold [24,39].
	Increased stress related to community violence, and insufficient public services resulting in a poor physical environment in low-income neighborhoods [39,42].

Insurance status	Association between lack of insurance or Medicaid and discharge against medical advice and worse asthma control [26,31].
	Emergency rooms as last resort for patients without health insurance in U.S. [28].

Race/ethnicity	Race/ethnicity as a marker for social disadvantage (e.g. low income, lack of insurance coverage) rather a risk factor itself [15,28,29].
	Association between black race/ethnicity and increased emergency room visits [28].
	Potential link between stress associated with multiple race identification and higher rates of asthma among adults who are both American Indian/Alaska Native and white [37].

Socioeconomic status	Relationship between race/ethnicity and discharge against medical advice neutralized when socioeconomic factors taken into account [31].
	Association between low socioeconomic status or lack of employment and increased asthma symptoms, poor compliance, and worse asthma-related quality of life [29,33,34,38].
	Asthma as both a consequence and contributor to poverty by restricting ability to work and incurring medical expenses [23,38].

The level of specificity in these pathways, and the strength of their implication of inequity, varied. In some cases, authors simply described elements of a pathway without considering their interconnections. We considered these elements in their social context to help us determine whether or not they were inequitable. For example, in a national-level study conducted in the United States, Rose et al [29] identified poverty as a risk factor for asthma and found that when poverty was included in their analysis, black Americans did not have significantly more asthma than white Americans. We considered this a discussion of inequity because findings of increased risk of asthma among black Americans should take into account their disproportionate risk of poverty and historic discrimination before attributing such differences to race. The authors did not go into detail, however, as to how lower SES may expose populations to more risk factors for asthma.

Other authors presented more detailed explanations of how social position may be translated into an increased risk of asthma. Thus, these authors implied more strongly that a given inequality was inequitable. Caldeira et al [23], for instance, found that adults with lower levels of education had a higher risk of developing work-related asthma. They pointed to possible connections between lower education, lower professional qualifications, and limited employment opportunities that may increase the likelihood of finding work in jobs with a higher exposure to environmental risk factors. In another example, Smith et al [30] found that asthma patients in the United States who were ambulance users tended to have lower levels of education. They suggested that a lack of access to public or private transportation among these populations may increase their likelihood of using ambulance services to seek medical care.

### Asthma-related inequalities

Asthma-related inequalities were described on the basis of SES, race/ethnicity, gender, place of residence, and/or health insurance status (for details see Additional file 1: Characteristics of included studies). Eighteen studies identified asthma-related inequalities by SES [15,23,24,26-40]. Studies generally identified greater prevalence or risk of asthma symptoms among populations with lower SES, and lower compliance [15,23,24,27,29,31,33,37-40]. With respect to health care utilization, lower SES was associated with increased use of emergency health services in two studies [26,28] and decreased use in one study [35].

Nine studies identified inequalities based on race/ethnicity [15,25-29,34,36,37]. All but one of these found that populations of certain races/ethnicities, particularly non-white populations, faced increased exposure to asthma-related risk factors and worse outcomes. The study by Hoffman et al [36], on the other hand, identified an association between white race/ethnicity and inconvenient clinic hours as a barrier to accessing health care for asthma. Seven studies identified gender-based inequalities [15,23,26,28,29,31,33]. Findings consistently identified a higher prevalence of asthma or asthma symptoms among women [15,23,29,33]. With respect to health care utilization, two studies found male gender to be associated with decreased risk of emergency care use for asthma [26,28].

Six studies identified asthma-related inequalities by place of residence [15,29,37,40-42], including worse asthma quality of life in neighborhoods with higher perceived problems [42], and increased asthma prevalence in residences with higher levels of pollutants or allergens [15,40]. Finally, three studies, conducted in the United States, identified inequalities based on health insurance status [26,31,37], such as increased rates of discharge against medical advice in asthma patients who were uninsured or had Medicaid as opposed to private insurance [31], and more asthma control problems among those with Medicaid versus private insurance [26].

### Interventions

Fifty-two percent (n = 11) of the studies suggested potential interventions stemming from their findings. Of these, 55% (n = 6) recommended exclusively clinical interventions [23,25,31,32,35,38]; that is, actions taken to improve the medical detection and/or management of asthma. They did not, however, suggest interventions to address underlying inequities producing disproportionate exposure to risk among certain groups or limiting ability to seek care. For example, Caldeira et al [23], mentioned above, identified an increased risk of work-related asthma among populations with low educational levels and hypothesized that this was linked to more limited and riskier employment opportunities available to this population. The only intervention suggested by these authors, however, was a targeted program of prophylaxis based on screening young workers for early symptoms of asthma. Similarly, Dixon et al [35] identified asthma and infrequent use of controller medication as an important problem among the Alaska Native population in the United States. The authors also highlighted that under-diagnosis of asthma may be an issue in this population if resident physicians are not available. They proposed better management of asthma through increased use of controller medications.

Three studies recommended interventions that address, at least partially, the upstream causes of the health disparities by targeting populations and environmental or social risk factors [26,40,42]. For instance, Yen et al [42] identified an association between perceived neighborhood problems and worse asthma-related quality of life. They suggested that the environment in low-income neighborhoods may contain more risk factors for asthma and that municipal governments may be less responsive to the populations in these neighborhoods. They proposed increased attention to public services such as trash removal, traffic calming measures, and regulation of emissions. In another example, Peters et al [26] proposed that the removal of racial and socioeconomic barriers could address disparities in asthma outcomes that were observed between patients with Medicaid insurance and those with private insurance.

The remaining two studies suggested both clinical interventions and interventions to address structural conditions. Alongside suggestions to treat asthma as a chronic disease, Hoffman et al [36] recommended more flexibility in clinic hours so that patients do not have to miss work to attend. Dimich-Ward et al [34] went further upstream to the level of public policy, and suggested that quality of life among those suffering from work-related asthma could be improved by better medical control in combination with retraining programs to facilitate work opportunities without exposure to aggravating factors.

## Discussion

This review identified 21 quantitative studies on asthma in adults that reported inequalities related to health outcomes or health service utilization. None of these studies referred to these inequalities as inequitable. However, we found that all but one included commentary that reflected underlying equity issues by attempting to trace pathways to inequity. This review did not include inequities related to methodological issues. While authors have previously considered this topic [43,44], we concentrated on discussions of equity related to research findings and implications for proposed interventions.

A more thorough examination of the inequity dimensions of asthma would be consistent with a social epidemiology approach focused on social structures that may increase exposure to causal factors. For example, does the geographic location of low SES neighborhoods persistently expose them to high diesel emissions from nearby trucking routes? The general absence of explicit consideration of equity in these articles raises concerns about their ability to provide evidence to inform interventions aimed at structural disadvantages (e.g. poverty) as a complement to clinical interventions. It may also hinder researchers from either moving beyond an emphasis on health service utilization to the upstream conditions that shape such utilization patterns, or finding an appropriate balance between health care aimed at treating those who are sick, and preventative measures designed to enhance and maintain health.

### Terms used

In addition to finding no occurrences of the term 'equity' in the eligible studies, this review identified only two articles that used 'inequality' and five that used 'disparity'. The majority of the studies used less-specific terms, such as 'difference' to describe the health inequalities they identified. The issue of terminology, and its political and moral associations, is not new to population and public health. A well-known example is the labeling of health inequalities as 'variations' by the Thatcher government in Britain rather than the more politically-charged language of inequalities used in the Black Report [45,46]. Several authors have argued that the language used in discussing health inequities can impact understandings of results and how inequities should be measured and addressed [1,2]. Choice of terminology may also influence studies selected for relevance review when certain search terms are applied.

Those conducting quantitative health research need to remain vigilant to the underlying etiological questions that drive methods and measures. While these are important to answer questions of causality and to determine attribution, they also persistently push the field towards an examination of proximal rather than distal determinants. But, as Kreiger et al [5] remind us, distal does not mean unimportant since it is these distal and often structurally embedded determinants that make for persistent health disparities.

### Studying and measuring inequity

There are an increasing number of illustrative studies in which authors have demonstrated the types of hypotheses that need to be tested when pathways to health equity are examined. With a better understanding of contextual influences, a more thorough examination of the social mechanisms that may be producing health outcomes can be undertaken. This is necessary for the delineation of upstream interventions. As an example, Subramanian et al [7] demonstrated that a re-examination of census data, that had yielded seemingly contradictory correlations between literacy and race, required an understanding of the historical context of segregated schooling in the southern United States (the Jim Crow laws).

A recent review of studies on measurement issues in health policy identified improving and clarifying metrics related to health disparities as a key requirement for eliminating such disparities [47]. This suggests the need for a taxonomy that guides the measurement of health inequities. However, developing measures that adequately capture the moral component of health inequities has been a challenge [48,49]. Asada [48], proposed a three-step framework to assist researchers to measure health inequities that involves selecting: (1) a definition of equity; (2) appropriate strategies to operationalize this definition; (3) measures to quantify health information. As Asada readily admits, however, this framework leaves many questions unanswered and further work is still needed to develop and agree upon consistent measures of health inequities. An improved set of measurement approaches for inequities would advance the field, but it is important to recognize that this is a complex undertaking given the number of mechanisms in the pathway that may be shaping inequities and their specificity to different types of health conditions.

### Implications for policy and practice

A related concern is that the lack of explicit discussion of equity found in this review may have implications for policy and practice. This review found that only 52% of the studies proposed interventions stemming from their findings, and that of these, 55% proposed interventions that focused exclusively on the more proximal, clinical level. While such interventions are important, they are unlikely to address underlying circumstances restricting opportunities for health, or to benefit disadvantaged populations. For example, while populations exposed to dust or chemicals in the workplace can be screened for early detection of asthma and medical care, such interventions neither address the concern that these workers may have limited alternative job options due to a lack of formal education and training, nor remove the inequitable and systematic exposure to increased health risks faced by those of lower socioeconomic status.

It is possible, then, that a lack of integration of equity into quantitative studies may have important implications for the kinds of interventions that are proposed and adopted. Research is needed that brings together proximal and distal concerns so that findings are contextualized within an understanding of resource distribution patterns and the opportunities accessible to different social groups. Conceptualizing and effectively examining such complex contributors to health inequity may greatly benefit from the theoretical and methodological innovation brought by transdisciplinary approaches to population health. The resulting evidence could inform multi-level interventions [50] that combine population- and clinical-level action.

## Conclusions

This review, therefore, finds that quantitative studies on asthma in adults have not discussed inequalities using an explicit equity lens. Without a more in-depth and systematic examination of inequities underlying asthma prevalence, these studies may fail to provide the evidence required to inform interventions targeted at key levers in the pathways to inequity. Further development of taxonomies of equity dimensions and knowledge of how pathways to inequity operate are needed to assist researchers to provide such evidence to policy makers and practitioners.

## Competing interests

The authors declare that they have no competing interests.

## Authors' contributions

HLG, NE, AH, EKK, ONO, and JAB all made substantial contributions to the data collection and to preparing and revising manuscript drafts. HLG further contributed to data analysis, coordinated the project, and led the writing team. NE further conceived of the study, supervised its execution, and contributed to data analysis. All authors read and approved the final manuscript.

## Pre-publication history

The pre-publication history for this paper can be accessed here:

http://www.biomedcentral.com/1471-2288/11/104/prepub

## Supplementary Material

Additional file 1 **CharacteristicsOfIncludedStudies.pdf**. Table containing complete list of included studies, basic study information (authors, study type, design), and description of asthma-related inequalities in exposure or outcome variables. Click here for file
