# Supplementary material for: A review of equity issues in quantitative studies on health inequalities: the case of asthma in adults
Source: BMC Med Res Methodol. 2011 Jul 12;11:104. doi: 10.1186/1471-2288-11-104 (PMC3149599; doi:10.1186/1471-2288-11-104)
Supplement: Additional file 1 — CharacteristicsOfIncludedStudies.pdf. Table containing complete list of included studies, basic study information (authors, study type, design), and description of asthma-related inequalities in exposure or outcome variables. [file 1471-2288-11-104-S1.PDF]

Additional file 1. Characteristics of included studies and asthma-related inequalities in exposure or outcome variables

Authors (Year)	n	Study Type	Design	Socioeconomic Status	Race/ethnicity	Gender	Place of Residence	Insurance Status
Arif & Shah [27]	550	Etiological/ risk factor	Cross-sectional	Proportion of subjects reporting no wheezing attacks in last 12 months: (p=0.008)<br>Above poverty level:82.2%<br>Below poverty level: 9.1%	Exposure to dichlorobenzene by race/ethnicity (95%CI):<br>Mexican-American: 3.73 $\mu g/m^3$ (1.63,8.51)<br>non-Hispanic Blacks: 2.64 $\mu g/m^3$ (1.03–6.74)<br>non-Hispanic Whites:<br>0.68 $\mu g/m^3$ (0.35-1.30)			
Baptist et al [31]	6,828	Health services	Retrospective cohort	Discharge against medical advice by income (95%CI):<br>Income>$40,000 = ref<br>Income<$20,000, OR=2.69 (1.16,6.26)<br><br>Re-admission with 30 days by income (95%CI):<br>Income>$40,000 = ref<br>Income<$20,000, variable adjusted HR=2.32 (1.39,3.88)		Discharge against medical advice by gender (95%CI):<br>Female = ref<br>Male, OR=1.69 (1.22, 2.32)<br><br>Re-admission with 30 days by gender (95%CI):<br>Female = ref<br>Male, variable adjusted HR=1.75 (1.32, 2.32)		Discharge against medical advice by insurance status (95%CI):<br>Private=ref<br>Medicaid, OR=1.82 (1.20, 2.74)<br>Uninsured, OR=2.17 (1.41, 3.32)
Berger et al [32]	301	Etiological/ risk factor	Retrospective cohort	Prevalence of workplace exacerbation of asthma by selected job types (95% CI):<br>Security guard or police:62%(46-75%)<br>Janitorial:61%(49-73%)<br>Garment/textile manufacturing:<br>50% (33-67%)<br>Health aide: 25% (14-40%)				

Braback et al [24]	1,247,038	Etiological/ risk factor	Retrospective cohort	Asthma without allergic rhinitis by SES in entire sample (95% CI):<br>High SES = ref<br>Low SES, OR=1.14 (1.11,1.17)<br><br>Asthma with allergic rhinitis by SES in entire sample (95% CI):<br>High SES = ref<br>Low SES, OR=0.96 (0.93,1.00)<br><br>Asthma with allergic among low SES by cohort (95% CI):<br>High SES = ref for each cohort<br>Low SES 1952-1961, OR=0.72 (0.53,0.82)<br>Low SES 1962-1971, OR=0.92 (0.86,0.99)<br>Low SES 1972-1977, OR=1.07 (1.01,1.14)		
Caldeira et al [23]	1,922	Etiological/ risk factor	Cross-sectional	Work-related asthma by years of education (95%CI):<br>≥12yrs = ref<br>9-11yrs, OR=3.41(1.64,7.10)<br>1-8yrs, OR=6.82 (2.99,15.57)		Proportion of subjects by gender with bronchial hyperresponsiveness ($p<0.0001$):<br>Female: 61.6%<br>Male: 38.4%
Corvalan et al [33]	1,232	Etiological/ risk factor	Cross-sectional	Wheeze & another asthma symptom by education (95% CI):<br>OR for each additional year of education=0.93(0.88,0.99)<br><br>Wheeze & another asthma symptom by domestic belongings(95% CI):<br>Domestic belongings 3-5=ref<br>Domestic belongings 0-2, OR=1.66 (1.16,2.39)		Proportion of subjects by gender with bronchial hyperresponsiveness ($p<0.001$):<br>Female: 16.9%<br>Male: 8.0%

				Wheeze & another asthma symptom by car ownership (95% CI):<br>Car ownership ‘yes’=ref<br>Car ownership ‘no’, OR=1.08 (1.01,1.15)<br><br>Wheeze with atopy by overcrowding (95% CI):<br><1 = ref<br>>1.5, OR=0.53 (0.25,0.93)			
Dimich-Ward et al [34]	213	Etiological/ risk factor	Cross-sectional	Significant predictors of Quality-of-Life among subjects with Western Red Cedar Asthma [Beta coefficient(SE)]:<br>Unemployed: 0.9(0.3)	Significant predictors of Quality-of-Life among subjects with Western Red Cedar Asthma [Beta coefficient(SE)]:<br>Punjabi speaking: -0.6(0.3)		
Dixon et al [35]	3,197	Both	Prospective cohort	Emergency department visits and hospitalizations by income (95%CI):<br>Poverty income ‘no’=ref<br>Poverty income ‘yes’, OR=0.30 (0.10-0.91)			
Dumanovsky & Matte [15]	4,800	Etiological/ risk factor	Cross-sectional	Asthma prevalence by annual household income (poverty ratio), 95%CI:<br><100%=ref<br>600% or more, OR=0.32 (0.11,0.89)	Asthma prevalence by race/ethnicity ($p<0.001$):<br>Hispanics: 6.6%<br>Blacks: 5.9%<br>Whites: 3.3%<br><br>Current asthma by ancestry (95%CI):<br>Puerto Rican=ref<br>Dominican, non-US-born, OR=0.27 (0.18, 0.41)<br>Other Hispanic, non-US-born, OR=0.17 (0.11, 0.26)	Prevalence of current asthma by gender ($p<0.001$):<br>Female: 6.1%<br>Male: 3.2%<br><br>Asthma among Hispanics by gender (95% CI):<br>Female = ref<br>Male: OR=0.39 (0.28,0.56)	Asthma attacks in past month by housing environment (95%CI):<br>No cockroaches = ref<br>Cockroaches inside home, OR =1.71(1.08,2.71)<br><br>No rats or rodents = ref<br>Rats/rodents in neighborhood or building, OR=1.64 (1.06–2.54)
Haselkorn et al [25]	2,128	Both	Prospective cohort		Emergency department visits in past 3 months by race/ethnicity (95%CI):		

					White = ref<br>Black, OR=2.31 (1.46, 3.66)<br><br>Proportion with severe asthma by race (p=0.003):<br>White: 49.4%<br>Black: 60.9%			
Hoffmann et al [36]	34	Health services	Cross-sectional	Association between household income and ‘someone had to miss work’ as perceived barrier to health care: r=0.5, P=0.00<br><br>Association between being employed and perceived overall barriers to health care: t=2.80, P=0.00	Association between white race/ethnicity and ‘office hours not convenient’ as barrier to health care: t=2.30, P=0.02			
Iversen et al [41]	2,603	Etiological/ risk factor	Cross-sectional				Asthma prevalence by area of residence (95%CI):<br>Urban=ref<br>Rural, OR=0.59 (0.46, 0.76)	
Markovitz & Andresen [28]	2,195	Health services	Retrospective cohort	Urgent or emergency room care need in past year by employment status (95% CI):<br>Employed=ref<br>Unemployed/can’t work, OR=1.6 (1.2, 2.1)	Urgent or emergency room care use in past year by race/ethnicity (95% CI):<br>White/non-Hispanic=ref<br>Other/non-Hispanic race, OR=2.1 (1.4, 3.1)<br>Black/non-Hispanic race,OR=2.4 (1.7, 3.4)	Urgent or emergency room care need in past year by gender (95% CI):<br>Female=ref<br>Male, OR=0.6 (0.5,0.7)		
Peters et al [26]	1,315	Health services	Cross-sectional	Hospitalization or emergency department visit by education (95% CI):<br>Higher education=ref<br>Lower education,	Hospitalization or emergency department visit by race/ethnicity (95% CI):<br>White=ref	Hospitalization or emergency department visit by gender (95% CI):<br>Male=ref<br>Female=1.62		Asthma control problems by insurance status (95% CI):<br>Private=ref<br>Medicaid,

				OR=1.56 (1.14,2.15)	Race other than white, OR=2.08 (1.51–2.86)	(1.15,2.27)<br><br>Asthma control problems by gender (95% CI):<br>Male=ref<br>Female, OR=2.01 (1.61,2.50)		OR=1.67 (1.17,2.40)
Pleis & Barnes [37]	127,596	Etiological/ risk factor	Cross-sectional	Asthma diagnosis by poverty ratio (95% CI):<br>Poverty ratio <1.00 = ref<br>Poverty ratio 1.00-<2.00, OR=0.88 (0.80-0.96)<br>Poverty ratio greater or equal to 2.00, OR=0.76 (0.70-0.84)	Asthma diagnosis by race/ethnicity (95% CI):<br>White only=ref<br>American Indian/Alaska Native & white, OR=1.85 (1.45, 2.35)		Asthma diagnosis by region (95% CI):<br>West=ref<br>Northeast, OR=0.89 (0.82,0.96)<br>Midwest, OR=0.87 (0.81,0.94)<br>South, OR=0.82 (0.77,0.88)	Asthma diagnosis by insurance status (95% CI):<br>Uninsured=ref<br>Public, OR=1.28 (1.15,1.42)
Rose et al [29]	95,615	Etiological/ risk factor	Cross-sectional	Reported lifetime asthma by income (95%CI):<br>≥200% of poverty level=ref<br>100%-199% of poverty level,<br>OR=1.15 (1.05, 1.25)<br>below poverty level, OR=1.43 (1.31, 1.56)	Lifetime reported asthma by race/ethnicity (95%CI):<br>Non-Hispanic white=ref<br>Mexican, OR=0.56 (0.46, 0.69)<br>Mexican-American, OR=0.68 (0.59, 0.80)<br>Puerto Rican, OR=1.87 (1.49, 2.33)	Lifetime reported asthma by gender (95%CI):<br>Male=ref<br>Female, OR=1.32 (1.24,1.39)	Lifetime asthma by region (95% CI):<br>Northeast=ref<br>West, OR=1.33 (1.21, 1.46)<br><br>Lifetime asthma by area (95% CI):<br>Non-Metropolitan Statistical Area=ref<br>MSA, central city, OR=1.10 (1.02, 1.19)	
Smith et al [38]	133	Both	Cross-sectional	Association between current employment and clinician-assessed compliance (95%CI):<br>Poorly Compliant=ref<br>Compliant, OR=3.66 (1.00-13.44)<br><br>Association between receiving a larger # of welfare benefits and clinician-assessed compliance (95%CI):<br>Poorly Compliant=ref				

				Compliant, OR=0.56<br>(0.33,0.96)		
Smith et al [30]	142	Health services	Cross-sectional	Ambulance service use by education level (95%CI)<br>Year 12 and above=ref<br>Year 8 and below,<br>OR=4.36 (1.11-17.09)		
Tonorezos et al [39]	6,119	Etiological/ risk factor	Cross-sectional	Asthma prevalence by neighbourhood income (95%CI):<br>Low-income neighbourhoods: 5.8%<br>(4.6, 7.0)<br>Middle- to high-income neighbourhoods: 4.1%<br>(3.3, 4.8)		
Wilson et al [40]	31,704	Etiological/ risk factor	Cross-sectional	Asthma symptoms by education level (95% CI):<br>Education<senior high school=ref<br>Higher education,<br>OR=0.68 (0.53,0.87)<br><br>Asthma symptoms by occupational exposures (95% CI):<br>No exposure to gas=ref<br>Exposure to gas,<br>OR=1.56 (1.06,2.28)<br><br>No exposure to dust=ref<br>Exposure to dust,<br>OR=1.61(1.16,2.24)		Asthma risk by residential air pollution index (95%CI):<br>0=ref<br>1, OR=1.67<br>(1.15,2.42)<br>2, OR=2.68 (1.84, 3.91)<br>3, OR=3.27<br>(2.12,5.13)<br>4, OR=2.35<br>(0.64,8.81)
Yen et al [42]	435	Etiological/ risk factor	Cross-sectional			Asthma quality of life by perceived neighbourhood problem score, Beta(SE):<br>Quartile 1=ref<br>Quartile 3, 5.58(1.65),<br>P≤0.05

	Quartile 4<br>5.91(1.63), P≤0.05

Note: Blank cells indicate that authors did not assess or found no significant asthma-related inequalities for the variable in question.
